# Impact of Allostatic Load on Chronic Hepatitis B and C: A Narrative Review

**DOI:** 10.7759/cureus.92220

**Published:** 2025-09-13

**Authors:** Chukwuemeka E Ogbu, John N Kalu, Maureen Ezechukwu, Chinazor Umerah

**Affiliations:** 1 Internal Medicine, Cape Fear Valley Health, Fayetteville, USA

**Keywords:** allostatic load, biopsychosocial model, chronic hepatitis b (chb), chronic hepatitis c (chc), psychosocial stress

## Abstract

The clinical progression of chronic hepatitis B (HBV) and C (HCV) is highly variable and not fully explained by virological factors alone. This narrative review explores the role of allostatic load, defined as the cumulative physiological burden of chronic stress, as a host factor that may contribute to this heterogeneity by influencing immune dysregulation, hepatic inflammation, and clinical outcomes. We conducted a narrative synthesis of English-language studies identified through PubMed, Web of Science, Scopus, PsycINFO, and gray literature. We included studies (cross-sectional, case-control, cohort studies, clinical trials, implementation studies, and policy documents) that employed any measure of psychosocial stress (validated psychometric scales/biomarkers/clinical diagnoses) and hepatitis-related clinical or virologic outcomes. Preclinical studies that described biological mechanisms were also included. Given significant heterogeneity, findings were summarized thematically. Chronic stress disrupts hepatic immune homeostasis through neuroendocrine pathways, including the hypothalamic-pituitary-adrenal (HPA) axis and sympathetic nervous system activation, leading to Kupffer cell activation, pro-inflammatory cytokine release, and impaired antiviral immunity. Psychosocial stress and depression are associated with reduced adherence to antiviral therapy, particularly in HCV, and may influence disease flares and fibrosis progression. Structural and social determinants of health, such as poverty, trauma, adverse childhood experiences, and incarceration, impact allostatic load and create barriers to care, exacerbating health disparities in vulnerable populations. Allostatic load offers a biopsychosocial framework for HBV/HCV, but clinical evidence remains limited and heterogeneous, with a more established link in HCV. Future research should prioritize prospective studies using standardized stress biomarkers and evaluate integrated care models that address mental health and structural barriers to improve outcomes and achieve health equity.

## Introduction and background

Chronic hepatitis B (HBV) and hepatitis C (HCV) remain major global causes of cirrhosis and hepatocellular carcinoma (HCC) [1]. Yet even with effective antivirals and vaccination, clinical trajectories are strikingly heterogeneous. Some adults maintain low disease activity for years, whereas others progress rapidly to fibrosis or HCC [2,3]. This variability cannot be completely explained by viral factors, leading to the examination of host modifiers such as antiviral immune responses [4-6] and possible psychosocial stress exposures [7].

Allostatic load offers a useful lens to explain this variability. Introduced by McEwen and Stellar, it refers to the cumulative "wear and tear" that accrues as neuroendocrine and autonomic systems repeatedly adapt to stressors through the hypothalamic-pituitary-adrenal (HPA) axis and sympathetic nervous system [8,9]. Elevated allostatic load is a known risk factor for adverse outcomes across numerous chronic diseases [10], leading to the hypothesis that it may also impair immune surveillance and hepatic resilience in the context of viral hepatitis [11-14].

Evidence linking allostatic load to hepatitis outcomes is scattered across various disciplines, with preclinical and animal studies not fully translated into clinical practice. The impact of structural stressors such as poverty, stigma, and housing instability on disease progression is also underexplored. Therefore, this narrative review aims to synthesize this interdisciplinary evidence to examine the impact of allostatic load on chronic viral hepatitis. We explore biological pathways by which chronic stress can disrupt hepatic immune homeostasis and promote liver injury, appraise clinical evidence associating psychosocial stress with disease activity, treatment adherence, and outcomes, and consider social determinants that amplify stress burden and constrain care. By bringing this evidence together, we aim to highlight chronic stress as an underrecognized driver of HBV/HCV outcome variability and proffer priorities for future studies and integrated models of care.

## Review

Review design, search strategy, and data sources

This is a narrative review that aims to provide a comprehensive, thematic synthesis of interdisciplinary evidence rather than a strictly exhaustive and quantitative summary of the literature. We performed a targeted literature search of PubMed, Web of Science, Scopus, and PsycINFO. We screened the gray literature from the official repositories of the World Health Organization (WHO), the U.S. Centers for Disease Control and Prevention (CDC), and the U.S. Department of Health and Human Services (HHS). The search covered the period from January 2000 to May 2025 and was restricted to articles published in English. Boolean search strings were constructed to combine terms from four key domains: measures of allostatic load (cortisol, catecholamines, heart-rate variability, C-reactive protein (CRP), and cytokines); psychological measures (Perceived Stress Scale (PSS), Patient Health Questionnaire (PHQ-9), and Generalized Anxiety Disorder (GAD-7)); hepatitis (HBV and HCV); and hepatitis endpoints (liver enzymes, hepatitis flare, liver fibrosis, sustained virologic response (SVR), treatment initiation, and treatment adherence). The reference lists of all included articles were also hand-searched to identify any additional relevant publications. We included systematic reviews, observational cohorts, case-control, cross-sectional studies, clinical trials, and implementation studies of integrated care and preclinical and experimental animal studies for the mechanism. Given the significant heterogeneity in studies, the focus of which is a narrative review, a thematic synthesis was conducted to examine the literature around biological mechanisms of stress-induced immune dysregulation, clinical associations with disease activity and virologic outcomes, impact on treatment-related behaviors like adherence and completion, and the role of social determinants of health.

Conceptual framework

**Figure 1:**
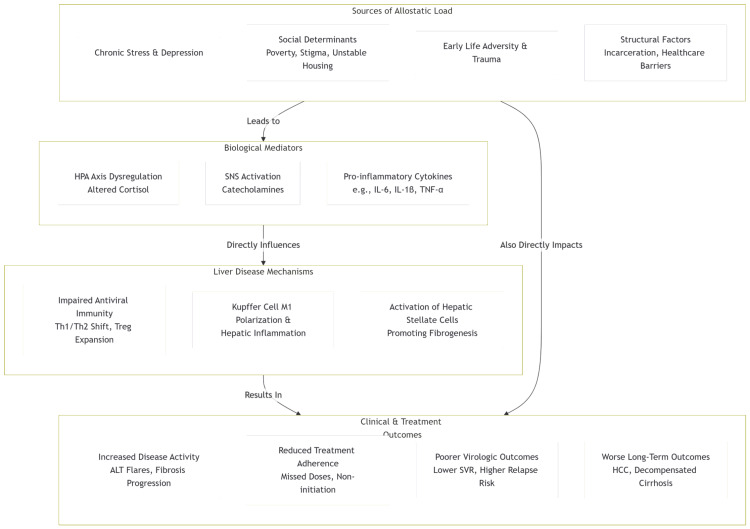
Conceptual framework Notes: Solid arrows indicate hypothesized direction of effect; side arrows labeled "Also Directly Impacts" denote pathways whereby upstream social/structural stressors can influence liver mechanisms and clinical outcomes independent of intermediate biological mediators. Examples in each box are illustrative, not exhaustive. HPA = hypothalamic–pituitary–adrenal; SNS = sympathetic nervous system; IL = interleukin; TNF-α = tumor necrosis factor-alpha; Th1/Th2 = T-helper type 1/2; Treg = regulatory T cell; ALT = alanine aminotransferase; SVR = sustained virologic response; HCC = hepatocellular carcinoma; HSC = hepatic stellate cell; M1 = classically activated (pro-inflammatory) macrophage; KC = Kupffer cell. Figure created by the authors.

In this framework, social and structural stressors drive chronic psychosocial stress, which elevates allostatic load; this physiological dysregulation then directly mediates poorer hepatic and virologic outcomes. Figure 1 presents the conceptual framework of the study.

Chronic stress and neuroimmune disruption in liver disease

**Figure 2:**
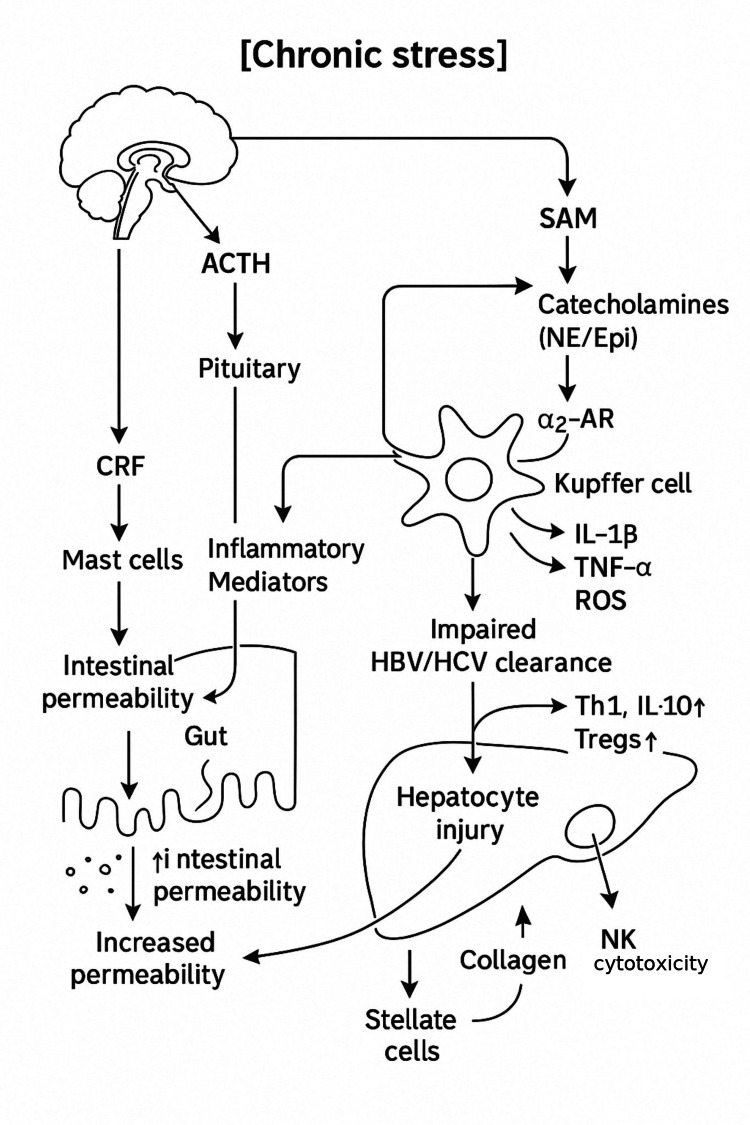
Chronic stress and neuroimmune disruption in chronic hepatitis Schematic of how chronic stress may promote injury in chronic HBV/HCV. Hypothalamic–pituitary–adrenal (HPA) and sympathetic–adrenal–medullary (SAM) axis activation modulates Kupffer cell function via adrenergic signaling, increasing pro-inflammatory cytokines (e.g., IL-1β, TNF-α) and ROS. Stress also increases intestinal permeability (via CRF and mast cells), permitting portal translocation of LPS and TLR4 engagement on Kupffer cells. These pathways contribute to impaired antiviral clearance (↓Th1, ↑IL-10, Treg expansion), reduced NK-cell cytotoxicity, hepatocyte injury, and fibrosis. ACTH = adrenocorticotropic hormone; CRF = corticotropin-releasing factor; HPA = hypothalamic–pituitary–adrenal; HCC = hepatocellular carcinoma; LPS = lipopolysaccharide; NK = natural killer; ROS = reactive oxygen species; SAM = sympathetic–adrenal–medullary; TLR4 = toll-like receptor 4; Treg = regulatory T cell. Figure created by the authors as synthesized from the literature review.

Chronic stress affects liver physiology through different neuroimmune pathways (Figure 2).

Under physiologic conditions, the liver maintains immune tolerance by filtering gut-derived antigens while suppressing unnecessary immune responses through tolerogenic Kupffer cell signaling, including interleukin-10 (IL-10) secretion. However, chronic stress disrupts this balance through neuroendocrine activation of the HPA axis and sympathetic-adrenal-medullary (SAM) system, leading to the release of glucocorticoids and catecholamines, which reprogram immune cell activity and inflammatory cytokine profiles.

Evidence from experimental studies suggests that stress interferes with hepatic immune tolerance, leading to sustained inflammation and tissue injury [15-23]. In animal studies, acute psychological stress was shown to reduce hepatic blood flow by up to 60% via central corticotropin-releasing factor (CRF) signaling, resulting in hypoxic-reoxygenation injury that triggers mitochondrial reactive oxygen species (ROS) production and inflammatory cytokine release by Kupffer and endothelial cells [15-18]. These hypoxic conditions deplete hepatic ATP, causing hepatocyte necrosis and further worsening hepatic inflammation. Kupffer cells are key mediators of stress-induced liver injury, typically involved in immune tolerance, and are susceptible to stress signals. Restraint stress models have shown that Kupffer cells, when stimulated by necrosis-derived damage-associated molecular patterns (DAMPs) such as Interleukin-1 alpha (IL-1α) and extracellular mRNA, activate proinflammatory pathways like Nuclear Factor Kappa-Beta (NF-κB) that leads to secretion of IL-1β and ROS [19-22]. This drives the recruitment of neutrophils and further increases liver injury. Moreover, Kupffer cell polarization toward an M1 proinflammatory phenotype also disrupts hepatic immune tolerance and fosters a microenvironment conducive to chronic inflammation and fibrosis [23].

In another downstream mechanism, chronic stress increases intestinal permeability via CRF- and acetylcholine-mediated mast cell activation, allowing the translocation of gut-derived lipopolysaccharide (LPS) into the portal circulation [24,25]. LPS influx further polarizes Kupffer cells toward a proinflammatory state [25]. At the same time, norepinephrine released from the gut during ischemia-reperfusion induces robust TNF-α secretion from Kupffer cells, a response blocked by α2-adrenergic antagonism [26-28]. There is also a skewed systemic and local immune response in ways that may impair viral control and facilitate oncogenesis [14]. Sustained elevation of glucocorticoids can cause functional exhaustion of cytotoxic T lymphocytes and natural killer cells, while favoring expansion of immunosuppressive cell types [14]. In chronic HBV infection (CHB), an effective antiviral immune response (Th1-cytotoxic) is needed to control the virus, but chronic stress may tilt the balance toward immune tolerance or exhaustion [28].

One clinical study found that patients with CHB who experienced high psychosocial stress had unexpectedly low levels of hair cortisol [29]. This paradoxical suppression of cortisol was associated with impaired immune function and a potentially diminished infection control ability [29]. Animal studies have shown that chronic stress alters leukocyte trafficking and the splenic immune cell composition, reducing the frequency of anti-tumor effector cells and increasing Tregs [14]. These findings collectively support a dual model wherein chronic stress amplifies hepatic inflammation through Kupffer cell overactivation and oxidative stress and simultaneously weakens antiviral defenses through Th2 polarization and Treg expansion. This "allostatic overload" fosters an internal hepatic microenvironment that promotes fibrosis and immune tolerance, which are factors involved in chronic hepatitis infection. While these findings primarily derive from preclinical and animal models, they offer a hypothetical framework for understanding how chronic stress may act together to promote immune suppression and hepatic injury in chronic viral hepatitis.

Stress, depression, and liver disease activity in HBV/HCV

Cross-sectional studies have associated psychosocial stress and depression with the severity of chronic hepatitis infection. Nagano et al. (2004) reported higher chronic psychosocial stress and a "type 1" stress-prone personality as associated with higher severity of HCV infection [30]. Psychosocial stress was measured with the 45-item Stress Inventory (SI), which yields scales related to the type-1 personality (low sense of control, object-dependence of loss, unfulfilled need for acceptance, and altruism). Liver disease severity was defined as categories (normal ALT, elevated ALT, and cirrhosis) based on laboratory and clinical diagnosis in the hepatology clinic. They found that higher scores on SI were strongly associated with severe chronic hepatitis C (CHC) infection after control of confounders. Their results were similar when interferon-treated patients were excluded, addressing the concern that prior therapy might have altered mood/stress measures.

Tsai et al. (2022) conducted a nationwide population-based retrospective cohort study using the Taiwan National Health Insurance database to determine the relationship between hepatitis B flares and depression or anxiety using propensity scoring and found that patients with clinically diagnosed depression or anxiety had a significantly higher incidence rate of hepatitis B flares than those without depression or anxiety [31]. This association was significant after adjusting for age and comorbidities. Shaheen et al. (2023) conducted a retrospective cohort study on HBV and HCV patients, assessing the impact of major depressive disorder and antidepressant use on outcomes like first decompensation or all-cause mortality. They found that an MDD diagnosis did not worsen clinical outcomes in these patients [32]. However, specific antidepressant classes were associated with increased risks. TCAs were associated with worse decompensation-free survival in HCV, while SSRIs were associated with higher mortality in HCV. In HBV, TCA use was also associated with increased mortality in sensitivity analyses. The study's limitations include an inability to account for antiviral treatments and potential residual confounding.

Cytokines and neuroendocrine signals in chronic HBV/HCV

Studies suggest that psychological stress can modulate hepatitis activity through immune pathways, though direct evidence linking stress to fibrosis progression in viral hepatitis has not been validated in prospective studies. The association also appears context-dependent. For example, He et al. (2014), in their cross-sectional study of 80 adults with CHB infection, found that patients with newly diagnosed depression or anxiety had higher rates of HBV flares as measured with ALT elevations during follow-up compared with propensity-matched controls. They also found that depression/anxiety remained a significant independent predictor after adjustment [7]. Stress was measured with the Perceived Stress Scale 10 (PSS-10), while anxiety was measured with the State-Trait Anxiety Inventory (STAI). The severity of HBV was measured with elevated blood levels of ALT, HBV DNA, lymphocyte subsets, IL-10, and IFN-γ in the peripheral blood. They found that higher stress and anxiety were associated with lower ALT levels and a significantly reduced IFN-γ:IL-10 ratio. Their finding suggests stress may acutely suppress cytotoxic inflammation. However, their use of a single time-point ALT is a less useful indicator of underlying disease activity in stressed patients.

However, converging psychoneuroimmunology evidence indicates that chronic stress promotes a pro-inflammatory and pro-fibrotic state. In hepatitis C cohorts, depressive symptoms correlate with elevated IL-1β and TNF-α, cytokines that directly mediate hepatocyte injury [33]. IL-6, which is a key stress-responsive cytokine, is a potent driver of hepatic stellate cell activation and fibrogenesis. One study showed that polymorphisms in the IL-6 pathway are associated with accelerated fibrosis in CHC, positioning it as a critical biological relay between sustained stress and liver matrix deposition [34,35]. A hypothesized pathway involves stress-exacerbated gut-barrier dysfunction, where subsequent LPS release activates TLR4 signaling, which is a well-established mechanism implicated in fibrosis progression across liver diseases [36].

Therefore, short-term stress may dampen cytolytic activity, explaining the inverse ALT correlation, while the long-term effect of maladaptive stress signaling through IL-6, IL-1β, TNF-α, and LPS-TLR4 likely creates a permissive environment for chronic inflammation and fibrogenesis [7,34-36]. This proposed mechanism is, however, hypothetical, and more prospective studies that associate serial, standardized stress measures with definitive clinical assessments are needed.

Impact on treatment adherence and virologic outcomes

It has been hypothesized that psychosocial stress burden shapes the response to treatment through behavioral pathways of adherence and persistence and potentially biologic modulation.

Historically, IFN-α treatment for HCV created a challenging situation. The treatment itself could cause severe depression, while pre-existing depression or anxiety significantly reduced a patient's likelihood of both initiating and completing the arduous treatment course. Liu et al. demonstrated that patients with pre-existing depression on prophylactic antidepressants had lower completion (52%), while those who developed depression during therapy and received on-demand treatment had higher completion (92%) [37]. The authors also report no significant relationship between antidepressant therapy and SVR among those who completed treatment, indicating that the primary barrier was behavioral (adherence and persistence) rather than biological [37].

In the Direct-Acting Antiviral (DAA) era, one study showed that although overall DAA adherence is high, early-phase non-adherence, which is a critical window for achieving a cure, disproportionately affects individuals with high allostatic load [38]. In the PREVAIL study, which used electronic blister packs to monitor adherence in people who inject drugs (PWID), total adherent doses and especially early adherence in weeks 1-4 and 5-8 were predictors of SVR, whereas adherence in the final month was not [38]. Moreover, alcohol intoxication, which is a correlate of psychosocial burden, was associated with erratic dosing, while drug use was not [38]. One randomized Veteran Affairs trial showed that integrated mental health/case management care roughly doubled treatment initiation and improved overall SVR across all randomized participants versus usual care [39].

For CHB, treatment involves long-term, often lifelong, daily adherence to oral nucleos(t)ide analogues (NAs) to maintain virologic suppression and prevent resistance. The literature directly linking measured psychosocial stress to NA adherence is very scarce. Large pharmacy claims analysis shows imperfect persistence (81%) and adherence (88%) patterns, with younger and new-start patients at greater risk for missed dosing [40]. Moreover, analyses of virologic breakthroughs have shown that a significant portion are not due to antiviral resistance but are instead attributable to intermittent non-adherence, often resolving without a change in therapy once adherence is addressed [41]. Adherence below 90% is a significant risk factor for breakthroughs, even on high-barrier agents like entecavir [42]. Population- and clinic-level cross-sectional studies link depression and anxiety with self-reported non-adherence and HBV-related stigma with lower antiviral adherence, aligning with a stress-adherence pathway, though HBV-specific evidence remains thinner than for HCV [43-45].

Therefore, while depression and anxiety are clinical comorbidities and not allostatic loads themselves, they are used as sustained psychosocial stress and plausibly contribute to AL through HPA/SNS activation and inflammatory signaling. Framed this way, psychiatric symptoms and stigma function as modifiable stress-burden markers that erode adherence in both HCV and HBV, most convincingly shown in interferon-era HCV and increasingly suggested for oral HBV therapy. In the DAA era, studies can use validated stress assessment tools such as the PHQ-9, PSS-10, and stigma scales in combination with objective adherence measures, including electronic monitoring, pharmacy refill records, and biomarker panels that quantify allostatic load. This approach also includes monitoring antiviral exposure and virologic outcomes (Table 1).

**Table 1:** Psychosocial stress, depression, adherence, and treatment outcomes in chronic hepatitis B and C: synthesis of selected articles HBV = hepatitis B virus; HCV = hepatitis C virus; CHB = chronic hepatitis B; CHC = chronic hepatitis C; NUC(s) = nucleos(t)ide analogue(s); LAM = lamivudine; ADV = adefovir dipivoxil; ETV = entecavir; TBV = telbivudine; TDF = tenofovir disoproxil fumarate; DAA(s) = direct-acting antiviral(s); PEG-IFN-α = pegylated interferon-alpha; RBV = ribavirin; SVR = sustained virologic response; VBT = virologic breakthrough; GR = genotypic resistance; BBT = biochemical breakthrough; ALT = alanine aminotransferase; HBeAg = hepatitis B e antigen; IL-6 = interleukin-6; MADRS = Montgomery–Åsberg Depression Rating Scale; BDI = Beck Depression Inventory; HAMD = Hamilton Depression Rating Scale; HAMA = Hamilton Anxiety Rating Scale; PSS-10 = Perceived Stress Scale-10; STAI = State-Trait Anxiety Inventory; MDD = major depressive disorder; CBT = cognitive behavioral therapy; MI = motivational interviewing; OAT = opioid agonist therapy; PWID = people who inject drugs; DOT = directly observed therapy; ASI = Addiction Severity Index; THIN = The Health Improvement Network (UK primary-care EHR); IFN = interferon; OR = odds ratio; HR = hazard ratio; aHR = adjusted hazard ratio; CI = confidence interval.

Infection	Authors (Year)	Study Title	Design, N	Stress/Psychosocial Measure	Treatment Era/Exposure Captured	Outcomes	Key Findings	Main Limitations
HCV	Nagano et al., 2004 [30]	Psychosocial Stress, Personality, and the Severity of Chronic Hepatitis C	Cross-sectional clinic study. N=51 (33 men, 18 women; mean age 45.5)	Stress Inventory (45 items). Type-1 personality composite.	Mixed, IFN-era. IFN-treated patients were excluded from the sensitivity analysis.	Hepatitis severity: A) Normal ALT, B) Elevated ALT, C) Cirrhosis (clinical diagnosis).	Higher stress/personality scores are associated with higher odds of elevated ALT/cirrhosis.	Cross-sectional; small sample; no fibrosis staging; no physiologic biomarkers.
HBV	He et al., 2014 [7]	Psychological Stress Exerts Effects on Pathogenesis of Hepatitis B via Type-1/Type-2 Cytokines Shift	Cross-sectional study. N=80	Perceived Stress Scale-10 (PSS-10), State-Trait Anxiety Inventory (STAI)	Not applicable (no treatment exposure measured)	ALT, HBV DNA, lymphocyte subsets, IL-10, IFN-γ	Higher stress/anxiety is associated with lower ALT and reduced IFN-γ:IL-10 ratio.	Cross-sectional; no histology; single-time ALT measure.
HBV	Tsai et al., 2022 [31]	Association Between Depression or Anxiety and the Risk of Hepatitis B Flare	Nationwide retrospective cohort. N=12,920 with depression/anxiety vs. 51,680 matched controls	Clinical diagnosis from insurance claims data.	Claims-based; exclusions for advanced liver disease at baseline.	HBV flare (ALT > 2x ULN + nucleos(t)ide analog prescription)	The depression/anxiety cohort had a higher cumulative incidence of flares (aHR=1.17).	Claims-based exposure; potential detection bias.
HBV & HCV	Shaheen et al., 2023 [32]	Impact of depression and antidepressant use on clinical outcomes of hepatitis B and C	Retrospective cohort. HBV (n=1,418), HCV (n=1,835). Median FU ~6.3y (HBV), ~5.2y (HCV)	Coded MDD and antidepressant use (>90 days supply).	Pre- and DAA era. Prescriptions before any decompensation event.	First decompensation event or all-cause death.	MDD diagnosis is not an independent predictor of outcomes. TCA use is associated with worse decompensation-free survival in HCV; SSRI is associated with higher mortality in HCV.	Indication bias; limited treatment detail.
HCV	Loftis et al., 2008 [33]	Depressive symptoms in patients with chronic hepatitis C are correlated with elevated plasma levels of interleukin-1β and tumor necrosis factor-α	Cross-sectional. HCV+ (n=16) vs. HCV- controls (n=7); Total N=23	Beck Depression Inventory-II (BDI-II)	Pre-DAA; IFN-naïve at enrollment.	Plasma IL-1β, TNF-α, IL-6; correlation with BDI-II.	The HCV group had higher TNF-α. Within HCV patients, higher BDI-II correlated with higher TNF-α and IL-1β.	Small, male-predominant VA sample; cross-sectional; no fibrosis staging.
HCV	Frydecka et al., 2016 [34]	Functional Polymorphism in the Interleukin 6 (IL6) Gene with Respect to Depression Induced in the Course of Interferon-α and Ribavirin Treatment	Prospective cohort on Peg-IFN-α/ribavirin; N=62	DSM-IV depressive episode (SCID), MADRS & BI.	Pre-DAA (Peg-IFN-α2a + ribavirin)	Incidence of IFN-induced depressive episode; symptom trajectory.	Baseline depressive symptoms predicted IFN-induced depression only among IL-6 G-allele (high-producer) carriers.	Small, single-center; no IL-6 levels; limited generalizability.
HCV	Liu et al., 2010 [37]	Impact of depressive symptoms and their treatment on completing antiviral treatment in patients with chronic hepatitis C	Retrospective single-center cohort; N=100 on IFN-based therapy	Clinically documented depressive symptoms; AD timing categories.	Pre-DAA (Peg-IFN + ribavirin)	Treatment completion; SVR; premature cessation.	On-demand AD was associated with the highest completion (92%) vs. prophylactic AD (52%); AD use did not affect SVR.	Small, retrospective; no standardized scale; not generalizable to DAA.
HCV	Pawłowski et al., 2024 [43]	The Severity of Depressive Symptoms as an Independent Predictor of Sustained Virological Response During Treatment of Hepatitis C	Prospective cohort; N=101 (SVR in n=99)	Montgomery–Åsberg Depression Rating Scale (MADRS)	Pre-DAA (Peg-IFN + ribavirin)	SVR at 24 weeks post-treatment.	Greater baseline depressive severity (MADRS) independently predicted lower odds of SVR (OR 0.88).	Interferon-era therapy; baseline depression measured once.
HCV	Heo et al., 2021 [38]	Hepatitis C Virus Direct-Acting Antiviral Treatment Adherence Patterns and Sustained Viral Response Among People Who Inject Drugs	RCT in OAT programs; N=113	BDI ≥ 20, psychiatric illness, alcohol intoxication (ASI), drug use.	DAA era (SOF/LDV; SOF/SIM); planned 8/12/24 weeks.	SVR vs. adherence patterns (weeks 1–4, 5–8, 9–12).	Higher overall and early-treatment adherence strongly predicted SVR; alcohol intoxication signaled irregular adherence.	Small; PWID only; 12-week adherence truncation; ingestion not confirmed.
HCV	Groessl et al., 2017 [39]	HCV Integrated Care: A Randomized Trial to Increase Treatment Initiation and SVR with Direct-Acting Antivirals	Single-center RCT: Integrated Care (IC) vs. Usual Care (UC); N=79	BDI, PC-PTSD, AUDIT-C, recent drug use/urine tox.	First-gen DAA era (Boceprevir/Telaprevir + Peg-IFN/RBV)	Treatment initiation (time-to-event) and SVR.	IC doubled initiation vs. UC (45% vs. 23%); higher ITT SVR in IC (30% vs. 13%; p=0.07).	Underpowered single-site VA trial in the first-gen DAA era.
HBV	Chotiyaputta et al., 2011 [40]	Persistence and adherence to nucleos(t)ide analogue treatment for chronic hepatitis B	Retrospective claims analysis; N=1,100	None	Oral NUCs (lamivudine, adefovir, entecavir, telbivudine)	Persistence (no gap >90 days) and adherence (MPR).	Persistence was higher among the elderly compared to new starts; adherence was 88%.	No stress measures; claims-based data.
HBV	Hongthanakorn et al., 2011 [41]	Virological Breakthrough and Resistance in Patients with Chronic Hepatitis B Receiving Nucleos(t)ide Analogues	Single-center retrospective cohort; N=168, mean FU 27.5 months	None	Various NUCs (lamivudine, adefovir, entecavir, telbivudine)	Virologic breakthrough (VBT) and genotypic resistance.	Many VBTs are not due to resistance and are resolved with continued therapy. Poor adherence is inferred as the cause.	No direct adherence data, regimen heterogeneity.
HBV	Kamezaki et al., 2013 [42]	Adherence to medication is a more important contributor to viral breakthrough in chronic hepatitis B patients treated with entecavir than in those with lamivudine	Single-center retrospective cohort; Entecavir (n=135) vs. Lamivudine (n=68)	Self-reported adherence	Entecavir vs. Lamivudine	Virologic breakthrough (VBT)	Poor adherence (<90%) strongly predicted VBT on entecavir; resistance was more prevalent on lamivudine.	Self-reported adherence, selection bias; single-center.
HBV	Zhu et al., 2016 [44]	Depression in patients with chronic hepatitis B and cirrhosis is closely associated with the severity of liver cirrhosis	Single-center prospective cohort; N=114 (Child-Pugh A/B/C)	Hospital Anxiety and Depression Scale (HADS)	Inpatient CHB receiving standard care; various NUCs.	HADS scores; prevalence of mood disorders by Child-Pugh class.	Depression/anxiety scores and prevalence rose with clinical severity (Child-Pugh C > B > A).	Hospitalized sample; cross-sectional; HADS-based categorization (no clinical interview).

Social determinants of allostatic load in chronic hepatitis infection

Specific high-risk populations for HBV and HCV infection also disproportionately experience chronic social stressors, contributing to elevated allostatic load that may worsen health outcomes (Figure 2).

**Figure 3:**
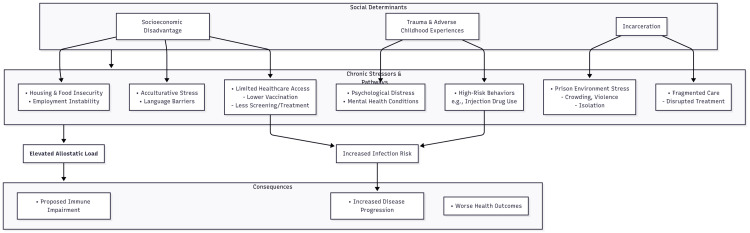
Conceptual pathway showing how social determinants drive chronic stressors in chronic HBV/HCV HBV = hepatitis B virus; HCV = hepatitis C virus. Figure created by the authors.

Socioeconomic disadvantage increases exposure to stressors such as housing insecurity, food scarcity, and limited healthcare access [46-51]. These factors contribute to higher rates of chronic viral hepatitis due to reduced vaccination, screening, and treatment [52,53]. Limited care and greater infection risk reinforce cycles of stress and disease progression [10,54,55]. In the United States, chronic HBV infection is more commonly observed among immigrant groups from endemic hepatitis regions, many of whom face language barriers, unstable employment, and limited insurance coverage [56]. These challenges can produce acculturative stress that adds to allostatic load and is linked to worse outcomes across chronic conditions [57]. Broader social inequities like community-level underinvestment, employment insecurity, and restricted access to specialty care can further increase the stress burden experienced by underserved populations [46,48,50]. Several practical strategies have demonstrated effectiveness. For example, expanding insurance coverage and eliminating treatment restrictions are linked with increased access to and uptake of hepatitis treatment [58]. Patient-centered and community-engaged interventions such as patient navigation, culturally tailored communication, and local partnerships have been shown to enhance linkage to care and retention rates [59-62].

Trauma and adverse childhood experiences (ACEs) are recognized as important contributors to allostatic load [63,64]. There is strong evidence linking ACEs to an elevated risk of HCV infection, predominantly through behavioral and social mechanisms. Individuals with a history of ACEs demonstrate a greater propensity to engage in high-risk behaviors, such as injection drug use, which continues to be the primary mode of HBV and HCV transmission in numerous contexts. A retrospective study involving liver transplant recipients indicated that those with HCV had a markedly higher prevalence of ACEs compared to recipients without HCV. This association was largely mediated by co-occurring substance use disorders and mental health conditions [65]. Cross-sectional studies of PWID have found a high prevalence of childhood adversity, which is linked to increased rates of adult medical and psychiatric comorbidities [66,67]. HBV and HCV status were not assessed in these studies. Direct longitudinal studies demonstrating that early trauma independently contributes to HBV/HCV disease severity or pathogenesis are currently unavailable, and all evidence is implied by other disease conditions. Nevertheless, the implementation of trauma-informed, integrated care models, where mental health and addiction services are embedded within hepatitis treatment, has been shown to increase treatment initiation and enhance overall cure rates in high-burden populations. These approaches also offer a practical means to address stress-related barriers to engagement [68]. Additionally, nurse-navigator programs for individuals with severe mental illness provide a scalable framework for such integrated care [59].

Incarceration is a salient social determinant for HBV/HCV. Approximately 15% of U.S. incarcerated persons are HCV-seropositive and 8-9% viremic [69]. HBV exposure is also markedly higher than in the general population [70]. Correctional environments impose chronic psychosocial stresses such as crowding, loss of autonomy, and violence, all of which augment allostatic load and can fracture care continuity [71]. Direct studies on immune suppression among incarcerated individuals with HBV or HCV are limited; however, comparisons can be made to studies on stress-related vaccine responses and chronic disease outcomes in high-stress environments [71]. Transitions into and out of incarceration often interrupt hepatitis care, resulting in missed opportunities for diagnosis, treatment, and follow-up. Effective models to address this crisis are emerging. The implementation of prison-based test-and-treat programs demonstrates high treatment completion and cure rates, with modeling confirming a significant public health benefit through reduced community transmission [72,73]. The challenge, however, remains the transition back to the community. Interventions like patient navigation, guaranteed appointments, and e-prescriptions are necessary to bridge the care gap, ensure patients continue treatment and achieve positive health outcomes.

Finally, allostatic load among individuals with HBV/HCV is shaped by social determinants. Populations such as disadvantaged communities, those with histories of trauma, and incarcerated individuals experience complex, overlapping stressors that negatively impact engagement with healthcare services and contribute to poorer health outcomes. Strategies to address these challenges include expanding healthcare coverage and implementing policy reforms [58], developing community-driven and culturally appropriate interventions [61], integrating trauma-informed behavioral health services, and establishing carceral test-and-treat programs with robust re-entry support [72,73]. These measures offer practical approaches to alleviating the impact of social determinants.

Future directions and interventions

One primary goal for the future is to advance beyond the use of general AL scores by developing a specialized toolkit calibrated for hepatitis. Infection-related inflammation may mimic "stress biology," which can confound measurement. Therefore, a hypothetical hepatitis-specific AL assessment should prioritize repeated evaluations of HPA-axis function, such as hair or salivary cortisol with diurnal slope analysis, DHEA-S, and the cortisol-to-DHEA-S ratio, in addition to ANS markers like resting heart rate, heart-rate variability, and catecholamines. Inflammatory biomarkers (CRP, IL-6, TNF-α) also require careful assessment [10,74-77]. The ultimate goal would be to combine these into a multi-system composite score, which is the gold standard for measuring allostatic load. This biomarker panel should then be integrated with key virus-specific clinical outcomes that are measurable.

On the clinical intervention side, integrated mental health and addiction care models and patient navigation models are actionable goals. Embedding behavioral health and case management into hepatitis care has been associated with increased treatment initiation and higher cure rates in high-stress populations. Nurse-navigator/community-health-worker programs can be trialed for patients with severe mental illness or unstable housing, with co-primary outcomes of treatment initiation, adherence, and AL change [59,60,68]. Adjunctive cognitive-behavioral therapy or mindfulness-based stress reduction merits practical evaluation in patients with elevated PHQ-9/PSS-10 scores. The feasibility of this approach is supported by studies that show that these methods can significantly alleviate psychological distress and may positively influence treatment adherence and clinical outcomes [78]. Lifestyle interventions such as aerobic exercise have also demonstrated beneficial effects. A study by El-Kader et al. reported that a three-month aerobic exercise program led to significant reductions in liver enzymes (ALT/AST) and improvements in mood states among patients with CHC [79]. Although specific studies on relaxation practices in HBV patients are limited, it is uncertain if these non-pharmacologic methods can be extrapolated to this HBV group. At a systems level, policy changes such as coverage expansion and removal of treatment restrictions have already improved DAA access. Equally important aspects include implementing prison-based test-and-treat programs with structured re-entry linkage to bridge a high-stress care transition and employing community-engaged, culturally tailored communication to reduce stigma and improve engagement in HBV care [80,81].

Further, exploration of stress-axis pharmacology may be worthwhile. Since the SNS acts via β-adrenergic receptors, it is postulated that non-selective β-blockers (NSBBs) may provide a targeted way to modulate this mechanism. Observational studies associate NSBB use in cirrhosis with lower rates of HCC [82] and improved survival [83], and these align with preclinical studies that show their anti-tumor effects [84,85]. The next step is a target-trial emulation in HBV/HCV cirrhosis cohorts to estimate the causal effect on HCC and survival, adjusting for confounders and immortal time bias. The aim is not to reduce overall allostatic load but to test SNS blockade as a therapeutic approach. Finally, AL remains a research tool and a conceptual lens for care planning. It is therefore not a definitive biomarker for clinical decisions in HBV/HCV. In the future, it may serve to stratify risk for poor treatment adherence, guide resource allocation toward supportive interventions, and physiologically quantify the burden of stress in patients with chronic hepatitis or diseases.

## Conclusions

The progression of chronic HBV and HCV is profoundly influenced by the psychosocial environment and can be mediated through the physiological pathways of allostatic load. Beyond the direct virological insult, chronic stress contributes to disease pathogenesis via neuroimmune dysregulation, activating Kupffer cells, promoting fibrogenic pathways, and impairing viral clearance. This burden is disproportionately borne by marginalized populations, where social determinants like poverty, trauma, and incarceration converge to create a syndemic of high viral prevalence and elevated allostatic load. The evidence base is currently stronger for HCV, and the proposed mechanisms are highly plausible for HBV and warrant further investigation. While preclinical models provide strong mechanistic evidence, translating these insights into clinical practice is not practicable and requires validating hepatitis-specific biomarkers of allostatic load and implementing integrated care models that address these psychosocial stressors. Ultimately, improving outcomes in viral hepatitis necessitates moving beyond a purely virological framework to embrace a biopsychosocial model of care that treats the whole patient, not just the virus.
